# Updated fiducial distribution of parameters in the associated delta-lognormal population

**DOI:** 10.1371/journal.pone.0298307

**Published:** 2024-06-05

**Authors:** Yufan Wang, Xingzhong Xu

**Affiliations:** 1 School of Mathematics and Statistics, Beijing Institute of Technology, Beijing, China; 2 School of Mathematics and Statistics, Shenzhen university, Shenzhen, China; Villanova University, UNITED STATES

## Abstract

In this paper we consider a special kind of semicontinous distribution. We try to concern with the situation where the probability of zero observation is associated with the location and scale parameters in lognormal distribution. We first propose a goodness-of-fit test to ensure that the data can be fit by the associated delta-lognormal distribution. Then we define the updated fiducial distributions of the parameters and establish the results that the confidence interval has asymtotically correct level while the significance level of the hypothesis testing is also asymtotically correct. We propose an exact sampling method to sample from the updated fiducial distribution. It can be seen in our simulation study that the inference on the parameters is largely improved. A real data example is also used to illustrate our method.

## 1 Introduction

In real applications, such as fisheries research and medical cost analysis, the response variables may be skewed, non-negative and have a non-negligible probability of zero outcomes. These variables are also known as following the semicontinous distribution

$$\begin{array}{c} F\left(x;\delta \right)=\{\begin{array}{cc} \delta , & x=0,\\ \delta +(1-\delta )G(x), & x>0, \end{array} \end{array}$$

where *G*(*x*) is the cumulative distribution of a postive variable. In existing researches, *δ* is usually assumed to be independent of *G*(*x*). However, we think that the probability *δ* is associated with *G*. For example, consider the precipitation distributions of some certain areas, the areas with larger rainfall per year are more likely to have less dry days. Hence, we can assume that *δ* is associated with *G*, say *δ* = *G*(*a*), for some *a*. In this paper, we try to deal with such assumption by a specified distribution, the delta lognormal distribution. This kind of distributions is first discussed and named by [1]. The cumulative distribution function of delta-lognormal distribution is then defined as follow

$$\begin{array}{c} F\left(x;\delta ,\mu ,\sigma \right)=\{\begin{array}{cc} \delta , & x=0,\\ \delta +\left(1-\delta \right)F_{LN}\left(x;\mu ,\sigma \right), & x>0, \end{array} \end{array}$$

where

$$\begin{array}{c} F_{LN}\left(x;\mu ,\sigma \right)=\Phi (\frac{\text{log}\;x-\mu }{\sigma }),x>0, \end{array}$$

denotes the cumulative distribution function of a lognormal distribution. Since log *X* follows a normal distribution *N*(*μ*, *σ*), we still refer to *μ* and *σ* as the location and scale parameters respectively in the rest of our paper.

[2] applied this distribution to deal with the measurement of worker exposure to air contaminants in United States. The use of delta-lognormal distribution to fisheries research was done by [3–5]. They considered the estimates of the population mean of the delta-lognormal distribution and further studied their robustness. It is easy to calculate the mean of the delta-lognormal distribution as

$$\begin{array}{c} M=\left(1-\delta \right)\;\text{exp}\left(\mu +\sigma^{2}/2\right). \end{array}$$

Much attention is given to the confidence interval of *M* by various statisticians. [6] proposed to use the likelihood ratio test to get a better control of the Type I error than the former standard ANOVA F-test and Kruskal-Wallis test. A Bootstrap approach is proposed which is proved to be second-order accurate in [7]. [8] considered the case when at least two non-zero observations are observed and modified the profiled loglikelihood function. [9, 10] used the generalized pivotal quantities proposed by [11] to construct a generalized pivot for estimating the mean. In their paper, a Beta distribution is used as the generalized pivot for *δ*. This thought is further developed by [12–14]. In the papers mentioned above, generalized pivot quantities are proposed for the binomial variable, which is discrete. Meanwhile, the conclusion of [15] on generalized fiducial inference also motivates some new ideas. The recent results are shown in [14], where the authors focus mainly on the improvement of the Beta distribution to approximate the generalized fiducial distribution of *δ*.

Instead of finding a generalized fiducial distribution, another method is proposed by [16], named “method of variance estimates recovery”(MOVER). This method can be easily applied to many different settings while guarantees the coverage probability of the confidence interval. From the Bayesian perspective, [17] compared the performance of using different prior distributions for both lognormal distribution and delta-lognormal distribution. They further considered the comparison of the means of two lognormal population.

As we can see from the introduction above, the three parameters in delta-lognormal distribution is assumed to be independent. However, in real applications, the probability of zero outcomes may be associated with the location and scale parameters. Consider the case of the spend on children’s clothing in [1], a family in a rich community is more likely to be a spender, while the one in a relative poor community may be a nonspender, since it is easy to be influenced by other families in the same community. It is natural to assume that the probabiliy of the nonspender in a community with large *μ* and *σ* may be smaller than that of a community with small *μ* and *σ*. Similar cases illustrate that in real applications, *δ* may depend on the other two parameters. We refer to this special kind of distribution as an associated delta-lognormal distribution. Thus, we can learn information about *μ* and *σ* from both the nonzero observations and the number of zero observations.

Assume that *δ* is a known function of *μ* and *σ*. The unknown parameters of associated delta-lognormal distribution thus become (*μ*, *σ*). In this paper we will give the fiducial distributions and infer on the parameters. The idea is that we first obtain the fiducial distributions from the nonzero observations and then update them using the number of nonzero observations which follows a binomial distribution whose success rate is *δ*(*μ*, *σ*). The approach of updating is motivated by the Bayes theorem. The fiducial distributions of (*μ*, *σ*) from the nonzero observations is regarded as the “prior distribution”, and is combined with the binomial distribution to get the “posterior distribution”, which is referred to as the updated fiducial distribution. We further infer on *μ*, *σ* and functions of them by this updated fiducial distribution. The updated fiducial distributions of (*μ*, *σ*) are not derived from some statistics which are asymptotically normal. The asymptotically results of fiducial distribution given by [15] are no longer applicable here. Coincidentally, the updated fiducial distribution is the posterior distribution under the prior 1/*σ*. We show that this updated fiducial distribution enjoys the Bernstein-von Mises theorem. Then we show that the marginal fiducial distributions of the parametric functions are asymptotic confidence distributions defined in [18]. Therefore, the confidence intervals of the parametric functions have asymptotically correct confidence levels. The significance levels of the hypothesis testings are also asymptotically correct. To deal with the computation, we employ the reject-sampling motivated by the approximate Bayesian computation method, see [19–21]. Though there are some more superior sampling methods, our method is still promising benifits from its simplicity and exactness. We show in simulation study that our inference can be largely improved, due to the combination of the continous and discrete data.

The rest of the article is organized as follows. In Section 2, we introduce the associated delta-lognormal distribution and propose the updated fiducial distribution of the parameters. We further present approaches of confidence interval estimation and hypothesis testing of the parameters. Their frequentist properties are also given. We conduct simulations in Section 3 and use a real data example to illustrate our method in Section 4. We give our conclusion in the last section.

## 2 Methodology: Associated delta-lognormal distribution

In the articles mentioned earlier, three parameters in delta-lognormal distribution are always assumed to be independent. In this section, we consider the case when *δ* is associated with *θ* = (*μ*, *σ*). We assume that delta is a function of the location and scale parameters, denoted by *δ*(*μ*, *σ*). This means that an observation in the sample generated from the distribution may be 0 with probability *δ*(*μ*, *σ*) and the nonzero observations should follow a lognormal distribution with parameters *μ* and *σ*, which is denoted by LN(*μ*, *σ*). The cumulative distribution function of the associated delta-lognormal population is

$$\begin{array}{c} G\left(x;\mu ,\sigma \right)=\{\begin{array}{cc} \delta (\mu ,\sigma ), & x=0,\\ \delta \left(\mu ,\sigma \right)+\left[1-\delta \left(\mu ,\sigma \right)\right]F_{LN}\left(x;\mu ,\sigma \right), & x>0, \end{array} \end{array}$$

where *F*_*LN*_(*x*; *μ*, *σ*) is the cumulative distribution function of LN(*μ*, *σ*).

A sample from this population is denoted by *X* = (*X*_1_, *X*_2_, ⋯, *X*_*n*_). We assume that *N*_0_ observations are zero while the rest *N*_1_ = *n* − *N*_0_ ones are nonzero. The likelihood function for the number of zero observations *N*_0_ can be given as

P(N0=n0)=(nn0)[δ(μ,σ)]n0[1-δ(μ,σ)]n1, (1)

where *n*_0_ is the observation of *N*_0_, *n*_1_ = *n* − *n*_0_.

### 2.1 Updated fiducial distribution

Without loss of generality, we assume that the first *N*_1_ observations are nonzero, while the rest are 0, that is, $X=(X_{1},X_{2},\cdots ,X_{N_{1}},0,\cdots ,0)$. Given *N*_1_ = *n*_1_, the nonzero observations $X_{1},X_{2},\cdots ,X_{n_{1}}$ are from *LN*(*μ*, *σ*). Let *n*_1_ ≥ 2. A log-transformation is made to the observations, *Y*_*i*_ = log *X*_*i*_ for *i* = 1, 2, ⋯, *n*_1_. Then the sample mean and variance

$$\begin{array}{c} \bar{Y}=\frac{1}{n_{1}}\sum_{i=1}^{n_{1}}Y_{i},\;S^{2}=\frac{1}{n-1}\sum_{i=1}^{n_{1}}{\left(Y_{i}-\bar{Y}\right)}^{2}, \end{array}$$

follow a normal and *χ*^2^(*n*_1_ − 1) distribution respectively, that is,

$$\begin{array}{c} \bar{Y}∼N(\mu ,\frac{\sigma^{2}}{n_{1}}),\;\frac{\left(n_{1}-1\right)S^{2}}{\sigma^{2}}∼\chi^{2}\left(n_{1}-1\right). \end{array}$$

Let *U* ∼ *N*(0, 1) and *V* ∼ *χ*^2^(*n*_1_ − 1) be two independent random variables. Then we have

$$\begin{array}{c} \bar{Y}=\mu +\frac{\sigma }{\sqrt{n_{1}}}U,\;\left(n_{1}-1\right)S^{2}=\sigma^{2}V. \end{array}$$

Given $\bar{Y}=\bar{y}$ and *S*^2^ = *s*^2^, then *μ* and *σ* can be regarded as the functions of *U* and *V*

$$\begin{array}{c} \mu =\bar{y}-\frac{\sigma }{\sqrt{n_{1}}}U,\;\sigma^{2}=\frac{\left(n_{1}-1\right)s^{2}}{V}. \end{array}$$

The joint distribution of (*U*, *V*) is

$$\begin{array}{c} \frac{1}{\sqrt{2\pi }}e^{-\frac{u^{2}}{2}}\frac{v^{\frac{n_{1}-1}{2}-1}}{\Gamma \left(\frac{n_{1}-1}{2}\right)2^{\frac{n_{1}-1}{2}}}e^{-\frac{v}{2}}. \end{array}$$

Then the joint distribution of (*μ*, *σ*) can be calculated as

$$\begin{array}{c} \pi^{F}\left(\mu ,\sigma |x_{obs}\right)=\frac{\sqrt{n_{1}}}{\sqrt{2\pi }\sigma }e^{-\frac{n_{1}{\left(\bar{y}-\mu \right)}^{2}}{2\sigma^{2}}}\frac{{\left(s^{2}\right)}^{\frac{n_{1}-1}{2}}{\left(n_{1}-1\right)}^{\frac{n_{1}-1}{2}}}{\Gamma \left(\frac{n_{1}-1}{2}\right)2^{\frac{n_{1}-1}{2}}}{\left(\frac{1}{\sigma^{2}}\right)}^{\frac{n_{1}-1}{2}-1+\frac{3}{2}}e^{-\frac{\left(n_{1}-1\right)s^{2}}{2\sigma^{2}}}, \end{array}$$ (2)

where $x_{obs}=\left(x_{1},x_{2},\cdots ,x_{n_{1}},0,\cdots ,0\right)$.

This means that the fiducial distribution of (*μ*, *σ*) is

$$\begin{array}{c} \mu |\sigma^{2}∼N(\bar{y},\frac{\sigma^{2}}{n_{1}}),\;\frac{1}{\sigma^{2}}∼\frac{\chi^{2}\left(n_{1}-1\right)}{\left(n_{1}-1\right)s^{2}}. \end{array}$$ (3)

If *n*_1_ < 2, we take

$$\begin{array}{c} \mu |\sigma ∼N\left(\bar{y},\sigma^{2}\right),\;\frac{1}{\sigma^{2}}∼\chi^{2}\left(1\right) \end{array}$$ (4)

where $\bar{y}=0$ when *n*_1_ = 0 and $\bar{y}=y_{1}$ when *n*_1_ = 1. Then the fiducial density *π*^*F*^(*μ*, *σ*|*x*_*obs*_) in (2) is obtained for all *n*_1_ ≥ 0.

The fiducial distributions for lognormal distribution is first given by [22]. However, there is no common fiducial distribution for binomial variable. A generalized fiducial quantity is proposed by [15], which is a Beta distribution *Beta*(*n*_0_, *n*_1_ + 1). Other improvements made on the parameter of the Beta distribution is further proposed by [12, 14], which are 0.5[*Beta*(*n*_0_, *n*_1_ + 1) + *Beta*(*n*_0_ + 1, *n*_1_)] and *Beta*(*n*_0_ + 0.5, *n*_1_ + 0.5), respectively.

Now we consider the problem from the Bayesian perspective, without the need of using generalized fiducial quantities. In Bayesian inference, the prior beliefs about the model parameters *θ*, say *π*(*θ*), are updated by observing data *y*_*obs*_ through the likelihood function of the model. We denote the likelihood function by *p*(*y*_*obs*_|*θ*) and use the Bayes’ theorem to get the posterior distribution

$$\begin{array}{c} \pi \left(\theta |y_{obs}\right)=\frac{\pi \left(\theta \right)p\left(y_{obs}|\theta \right)}{\int_{\Omega }\pi \left(\theta \right)p\left(y_{obs}|\theta \right)d\theta }. \end{array}$$ (5)

The prior distribution is often specified by choosing some tractable distributions that we believe the parameters should obey. For associated delta-lognormal distribution, the prior distributions of (*μ*, *σ*) are naturally chosen to be the fiducial distributions (2), and is further updated by the likelihood function (1). We define the updated fiducial distribution of (*μ*, *σ*) as

πUF(μ,σ|Xobs)∝(nn0)[δ(μ,σ)]n0[1-δ(μ,σ)]n1×πF(μ,σ|Xobs), (6)

where “∝” means “proportion to”.

### 2.2 Goodness-of-fit test

Let the observation be *x*_1_, *x*_2_, ⋯, *x*_*n*_. We take *δ* = *G*(*x*_0_), where *x*_0_ is a preset value and *G* is the cumulative distribution function of the continuous part. In this paper, we consider the case when *G* is the lognormal distribution, then

$$\begin{array}{c} \delta \left(\mu ,\sigma \right)=\Phi (\frac{\text{log}\;x_{0}-\mu }{\sigma }). \end{array}$$

In real applications, *x*_0_ maybe known. For example, in Tobit model, see [23],

$$\begin{array}{c} f\left(x\right)=\{\begin{array}{ccc} & Y^{*},\; & Y^{*}>y_{\text{min}},\\ & 0,\; & Y^{*}\leq y_{\text{min}}, \end{array} \end{array}$$

then *x*_0_ = *y*_min_. When *x*_0_ is unknown, we can obtain *x*_0_ with the following method.

Let *n*_0_ and *n*_1_ be the numbers of zero and nonzero observations, respectively. Without loss of generality, let $x_{1},x_{2},\cdots ,x_{n_{1}}$ be the nonzero ones. Then *μ* and *σ* are estimated by

$$\begin{array}{c} \left\{ \begin{array}{cc} & \hat{\mu }=\frac{1}{n_{1}}\sum_{i=1}^{n_{1}}\;\text{log}\;x_{i},\\ & \hat{\sigma }=\sqrt{\frac{1}{n_{1}-1}\sum_{i=1}^{n_{1}}{\left(\text{log}\;x_{i}-\hat{\mu }\right)}^{2}}. \end{array}\right. \end{array}$$

Then

$$\begin{array}{c} \delta \left(\hat{\mu },\hat{\sigma }\right)=\Phi (\frac{\text{log}\;x_{0}-\hat{\mu }}{\hat{\sigma }}). \end{array}$$

Let $\delta \left(\hat{\mu },\hat{\sigma }\right)=n_{0}/n$, then

$$\begin{array}{c} \hat{x_{0}}=\text{exp}\;\{\hat{\mu }+\hat{\sigma }\Phi^{-1}(\frac{n_{0}}{n})\}. \end{array}$$

Thus, the associated delta can be given by

$$\begin{array}{c} \delta \left(\mu ,\sigma \right)=\Phi (\frac{\text{log}\;\hat{x_{0}}-\mu }{\sigma }). \end{array}$$

To test the goodness-of-fit, the classical Kolmogorov-Smirnov test is no longer suitable in the zero-inflated model. We consider using the Pearson’s chi-square test. The following partition is made on the internal [0, ∞), which is 0, (0, *a*_1_], (*a*_1_, *a*_2_], ⋯, (*a*_*k*_, ∞). Let

$$\begin{array}{cc} p_{0} & =\Phi (\frac{\text{log}\;x_{0}-\mu }{\sigma }),\\ p_{i} & =\Phi (\frac{\text{log}\;a_{i}-\mu }{\sigma })-\Phi (\frac{\text{log}\;a_{i-1}-\mu }{\sigma }),\;i=1,2,\cdots ,k, \end{array}$$

where *a*_0_ = 0. Then *p*_0_, *p*_1_, ⋯, *p*_*k*_ are estimated by

$$\begin{array}{cc} p_{0} & =\Phi (\frac{\text{log}\;x_{0}-\hat{\mu }}{\hat{\sigma }}),\\ p_{i} & =\Phi (\frac{\text{log}\;a_{i}-\hat{\mu }}{\hat{\sigma }})-\Phi (\frac{\text{log}\;a_{i-1}-\hat{\mu }}{\hat{\sigma }}),\;i=1,2,\cdots ,k, \end{array}$$

Let *m*_*i*_ be the number of samples in the interval (*a*_*i*_, *a*_*i*+1_), where *a*_*k*+1_ = ∞. We can then construct the following test statistic,

$$\begin{array}{c} T=\sum_{i=1}^{k}\frac{{\left(m_{i}-n{\hat{p}}_{i}\right)}^{2}}{n{\hat{p}}_{i}}+\frac{{\left(n_{0}-n{\hat{p}}_{0}\right)}^{2}}{n{\hat{p}}_{0}}. \end{array}$$ (7)

Then

$$\begin{array}{c} T\overset{d}{⟶}\chi^{2}\left(k+1-1-2\right)=\chi^{2}\left(k-2\right). \end{array}$$

Given the significance level *α*, the model of associated delta-lognormal distribution is accepted when

$$\begin{array}{c} T\leq \chi_{1-\alpha }^{2}\left(k-2\right). \end{array}$$

### 2.3 Inference on functions of parameters

Assume that (*μ*, *σ*) follows the updated fiducial distribution *π*^*UF*^(*μ*, *σ*|*x*_*obs*_). Let *G* = *g*(*μ*, *σ*) which is a random variable. Then we denote the marginal fiducial distribution of *g*(*μ*, *σ*) by $\pi_{G}^{UF}\left(g|x_{obs}\right)$ and the cumulative distribution function of $\pi_{G}^{UF}\left(g|x_{obs}\right)$ by $F_{G}^{UF}\left(g|x_{obs}\right)$.

#### Confidence interval

The confidence interval of *g*(*μ*, *σ*) with confidence level 1 − *α* is given by

$$\begin{array}{c} {}[{\hat{g}}_{\frac{\alpha }{2}}\left(x_{obs}\right),{\hat{g}}_{1-\frac{\alpha }{2}}\left(x_{obs}\right)], \end{array}$$ (8)

where ${\hat{g}}_{\gamma }\left(x_{obs}\right)$, 0 < *γ* < 1, satisfies

$$\begin{array}{c} F_{G}^{UF}\left({\hat{g}}_{\gamma }\left(x_{obs}\right)\right)=\gamma . \end{array}$$

#### Hypothesis testing

For the one-sided hypothesis

$$\begin{array}{c} H_{0}:g\left(\mu ,\sigma \right)\leq g_{0}\;versus\;H_{1}:g\left(\mu ,\sigma \right)>g_{0}, \end{array}$$

The *p*-value is defined as

$$\begin{array}{c} \hat{p}=\hat{p}\left(x_{obs}\right)=F_{G}^{UF}\left(g_{0}|x_{obs}\right). \end{array}$$ (9)

For the two-sided hypothesis

$$\begin{array}{c} H_{0}:g\left(\mu ,\sigma \right)=g_{0}\;versus\;H_{1}:g\left(\mu ,\sigma \right)\neq g_{0}, \end{array}$$

The *p*-value is then given by

$$\begin{array}{c} \hat{p}=2\;\text{min}\;\left\{\hat{p}\left(x_{obs}\right),1-\hat{p}\left(x_{obs}\right)\right\}. \end{array}$$ (10)

Now we start to investigate the frequenist properties of the confidence interval and the hypothesis testing. First we define the random variable *Z*_*i*_ as

$$\begin{array}{c} Z_{i}=\{\begin{array}{cc} 1, & X_{i}=0,\\ 0, & X_{i}>0, \end{array} \end{array}$$

where *i* = 1, 2, ⋯, *n*. Then (*Z*_1_, *X*_1_), (*Z*_2_, *X*_2_), ⋯, (*Z*_*n*_, *X*_*n*_) are independently identically distributed as *f*(*z*, *x*; *μ*, *σ*) given below. The population sample space is then X={0,1}×[0,∞) and the dominating measure ν=C×LN(0,1), where C is the counting measure on {0, 1} and *LN*(0, 1) is the standard log-normal distribution, which has the density as

$$\begin{array}{c} \frac{1}{x}\phi \left(\text{log}\;x\right),\;x\geq 0. \end{array}$$

When *x* = 0, we define the function above as the limit 0.

The density *f*(*z*, *x*; *μ*, *σ*) with respect to *ν* is

$$\begin{array}{c} f\left(z,x;\mu ,\sigma \right)=\delta^{z}{\left(1-\delta \right)}^{1-z}{\left[\frac{1}{\sqrt{2\pi }\sigma }\;\text{exp}\;(-\frac{{\left(\text{log}\;x-\mu \right)}^{2}}{2\sigma^{2}})/\phi \left(\text{log}\;x\right)\right]}^{1-z}, \end{array}$$ (11)

where (z,x)∈X, *θ* = (*μ*, *σ*) ∈ Ω = (−∞, ∞) × (0, ∞).

We first check that *f*(*z*, *x*; *μ*, *σ*) is a probability density function. It can be seen that when *Z* = 1, *X* = 0, the density is

$$\begin{array}{c} f(z,x;\mu ,\sigma )=f(1,0;\mu ,\sigma )=\delta , \end{array}$$

while when *Z* = 0, *X* = *x*, the density becomes

$$\begin{array}{c} f\left(z,x;\mu ,\sigma \right)=f\left(0,x;\mu ,\sigma \right)=\frac{1-\delta }{\sigma }\frac{\phi (\frac{\text{log}\;x-\mu }{\sigma })}{\phi (\text{log}\;x)}. \end{array}$$

Then we integrate *f*(*z*, *x*; *μ*, *σ*) on X with respect to *ν*

$$\begin{array}{cc} \int f(z,x;\mu ,\sigma )d\nu (z,x) & =\int_{0}^{+\infty }\delta \frac{1}{x}\phi \left(\text{log}\;x\right)dx+\int_{0}^{+\infty }\frac{1-\delta }{\sigma }\frac{\phi (\frac{\text{log}\;x-\mu }{\sigma })}{\phi (\text{log}\;x)}\frac{1}{x}\phi \left(\text{log}\;x\right)dx\\ & =\delta \int_{0}^{+\infty }\phi \left(y\right)dy+\frac{1-\delta }{\sigma }\int_{0}^{+\infty }\phi (\frac{y-\mu }{\sigma })dy\\ & =\delta +1-\delta \\ & =1. \end{array}$$

This indicates that *f*(*z*, *x*; *μ*, *σ*) is a density function with respect to *ν*.

Then we show that the family (11) is quadratic mean differentiable, which is defined below.

**Definition 1 (Quadratic Mean Differentiable)** *The family* {*P*_*θ*_, *θ* ∈ Ω} *is quadratic mean differentiable at* *θ*_0_ *if there exists a vector of real-valued functions* ${\dot{l}}_{\theta_{0}}$ *such that, as* *θ* → *θ*_0_,

$$\begin{array}{c} \int {\left[\sqrt{p_{\theta }}-\sqrt{p_{\theta_{0}}}-\frac{1}{2}{\left(\theta -\theta_{0}\right)}^{T}{\dot{l}}_{\theta_{0}}\sqrt{p_{\theta_{0}}}\right]}^{2}d\nu =o(\|\theta -\theta_{0}{\|}^{2}) \end{array}$$

To verify that a family is quadratic mean differentiable, a lemma below is used in this paper.

**Lemma 1** ([24]). *For every* *θ in an open subset of R*^*k*^, *let p*_*θ*_ *be the propbability density. Assume that the map* $\theta \mapsto s_{\theta }\left(x\right)=\sqrt{p_{\theta }}$ *is continuously differentiable for every x. If the elements of the Fisher information matrix I*_*θ*_ *are well defined and continuous in θ, then the density p*_*θ*_ *is quadratic mean differentiable*.

Hence we can establish the following proposition.

**Proposition 2** *Assume that* 0 < *δ*(*μ*, *σ*) < 1 *and δ*(*μ*, *σ*) *is continously differntiable for all* −∞ < *μ* < + ∞ *and σ* > 0. *Then the density f*(*z*, *x*; *μ*, *σ*) *is differentiable in quadratic mean*.

The proof of this propostion is given in S1 File.

Given the observation (*z*_1_, *x*_1_), ⋯, (*z*_*n*_, *x*_*n*_), we have the likelihood function as

$$\begin{array}{cc} \prod_{i=1}^{n}\;f\left(z_{i},x_{i};\mu ,\sigma \right) & =\delta^{n_{0}}{\left(1-\delta \right)}^{n_{1}}\prod_{i=1}^{n_{1}}[\frac{1}{\sqrt{2\pi }\sigma }\;\text{exp}\;(-\frac{{\left(\text{log}\;x_{i}-\mu \right)}^{2}}{2\sigma^{2}})/\phi \left(\text{log}\;x_{i}\right)]\\ & =\delta^{n_{0}}{\left(1-\delta \right)}^{n_{1}}\frac{1}{\sigma^{n_{1}}}\;\text{exp}\;[-\frac{1}{2\sigma^{2}}\sum_{i=1}^{n_{1}}{\left(\text{log}\;x_{i}-\mu \right)}^{2}]\;\text{exp}\;[\sum_{i=1}^{n_{1}}\frac{{\left(\text{log}\;x_{i}\right)}^{2}}{2}]. \end{array}$$

Notice that when *n*_1_ ≥ 2, the updated fiducial distribution has the form

πUF(μ,σ|xobs)∝(nn0)[δ(μ,σ)]n0[1-δ(μ,σ)]n1×πF(μ,σ|xobs)=(nn0)[δ(μ,σ)]n0[1-δ(μ,σ)]n1×n12πσexp[-n1(y¯-μ)22σ2]×(s2)n1-12(n1-1)n1-12Γ(n1-12)2n1-12(1σ2)n1-12-1×(1σ2)32exp[-(n1-1)s22σ2],

where *y* = log *x*. With simple calculation we can get

$$\begin{array}{c} \pi^{UF}\left(\mu ,\sigma |X_{obs}\right)\propto (\frac{1}{\sigma })\prod_{i=1}^{n}f\left(z_{i},x_{i};\mu ,\sigma \right). \end{array}$$

This means that the updated fiducial distribution can be regarded as a posterior distribution under the prior distribution 1/*σ*.

When *n* → ∞,

$$\begin{array}{c} P_{\theta }\left(N_{1}<2\right)=\delta {\left(\mu ,\sigma \right)}^{n}+n\delta {\left(\mu ,\sigma \right)}^{n-1}\left(1-\delta \left(\mu ,\sigma \right)\right)\to 0. \end{array}$$ (12)

Therefore we can apply the famous Bernstein-von Mises Theorem below to the updated fiducial distribution.

**Lemma 3 (Bernstein-von Mises Theorem**, [24]) *Let the experiment* (*P*_*θ*_ : *θ* ∈ Ω) *be differntiable in quadratic mean at θ*_0_ *with nonsigular Fisher information matrix* $I_{\theta_{0}}$, *and suppose that for every* *ε* > 0 *there exists a sequence of test* *ψ*_*n*_ *such that*

$$\begin{array}{c} E_{\theta_{0}}^{n}\psi_{n}\to 0,\;\underset{\|\theta -\theta_{0}\|\geq \varepsilon }{\text{sup}}\;E_{\theta }^{n}\left[1-\psi_{n}\left(Y\right)\right]\to 0. \end{array}$$

*Furthermore, let the prior measure be absolutely continuous in a neighborhood of* *θ*_0_ *with a continuous positive density at θ*_0_. *Then the corresponding posterior distributions satisfy*

$$\begin{array}{c} \|P_{\sqrt{n}\left(\theta_{n}-\theta_{0}\right)|X_{obs}}-N\left(\Delta_{n,\theta_{0}},I_{\theta_{0}}^{-1}\right)\|\overset{P_{\theta_{0}}^{n}}{\to }0. \end{array}$$ (13)

At the moment we explain notations in (13). The symbol $P_{\sqrt{n}\left(\theta_{n}-\theta_{0}\right)|X_{obs}}$ is the posterior density of $\sqrt{n}\left(\theta_{n}-\theta_{0}\right)$ while $N(\Delta_{n,\theta_{0}},I_{\theta_{0}}^{-1})$ is a normal distribution with mean

$$\begin{array}{c} \Delta_{n,\theta_{0}}=\frac{1}{\sqrt{n}}I_{\theta_{0}}^{-1}\frac{\partial l(\theta )}{\partial \theta }{|}_{\theta =\theta_{0}} \end{array}$$

and variance $I_{\theta_{0}}^{-1}$. The norm ‖*f* − *g*‖ means

$$\begin{array}{c} \int |f(x)-g(x)|dx, \end{array}$$

which is the *L*^1^ distance between densities *f* and *g*. Thus we can obtain the result below.

**Theorem 4** *Under the assumptions of Proposition 2, Bernstein-von Mises theorem holds when the posterior distribution is replaced by the updated fiducial distribution* *π*^*UF*^(*μ*, *σ*|*x*_*obs*_).

The proof of Theorem 4 is given in S1 File.

To explore the frequenist properties of the functions of parameters under updated fiducial distribution, we give the definitions of the confidence distribution and asympototic confidence distirbution, which were proposed by [18].

**Definition 2** *A function* *H*_*n*_(⋅) = *H*_*n*_(***X***_*n*_, ⋅) *on* X×Ω→[0,1] *is called a confidence distribution for a parameter* *θ* *if (i) for each given* $X_{n}\in X$, *H*_*n*_(⋅) *is a continuous cumulative distribution function; (ii) at the true parameter value* *θ* = *θ*_0_, *H*_*n*_(⋅, *θ*_0_) = *H*_*n*_(***X***_*n*_, *θ*_0_), *as a function of the sample* *X*_*n*_, *has the uniform distribution U*(0, 1). *The function* *H*_*n*_(⋅) *is called asymptotic confidence distribution if requirement (ii) above is replaced by (ii)*’ : *at θ* = *θ*_0_, $H_{n}\left(\theta_{0}\right)⇝U\left(0,1\right)$ *as n* → + ∞, *and the continuity requirement on H*_*n*_(⋅) *is dropped*.

The notation “⇝” means convergence in distribution.

Given *n*_1_ ≥ 2, under the fiducial distribution (2), it is well known that the marginal fiducial distributions of *μ* and *σ* are confidence distributions. However, under the updated fiducial distribution (6), the fiducial distributions (2) are updated by the discrete variable *N*_1_. Thus the marginal fiducial distributions are no longer confidence distributions. Except for *μ* or *σ*, we consider some functions of them. We have the following theorem.

**Theorem 5** *Let g*(*μ*, *σ*) = *K*(*aμ* + *bσ*), *where K is a strictly monotone increasing function. Then under the assumptions of Propostion 2, the marginal updated fiducial distribution of g is an asymptotic confidence distribution*.

The proof of Theorem 5 is given in S1 File.

Apply this theorem to different functions *g*(*μ*, *σ*), we can get the corollary below.

**Corollary 6** *The marginial updated fiducial distributions of the following functions are all asymptotic confidence distributions*:

(*i*) *g*_1_(*μ*, *σ*) = *μ*;
(*ii*) *g*_2_(*μ*, *σ*) = *σ*;
(*iii*) *g*_3_(*μ*, *σ*) = exp[*μ* + Φ^−1^(*γ*)*σ*], *the* *γ* *quantile of LN*(*μ*, *σ*);
(*iv*) *g*_4_(*μ*, *σ*) = Φ[(log *x*_0_ − *μ*)/*σ*], *the cumulative distribution fucntion of LN*(*μ*, *σ*) *at x*_0_;
(*v*) *g*_5_(*μ*, *σ*) = (1 − *δ*(*μ*, *σ*)) exp (*μ* + *σ*^2^/2), *the population mean, when*
  $$\begin{array}{c} \frac{1}{1-\delta }(-\frac{\partial \delta }{\partial \mu })+1>0. \end{array}$$ (14)

The proof of Corollary 6 is given in S1 File.

An example to (v) in Corollary 6 is *δ*(*μ*, *σ*) = Φ(−*μ*/*σ*). We can see that

$$\begin{array}{c} \frac{1}{1-\delta }(-\frac{\partial \delta }{\partial \mu })+1=1+\frac{1}{1-\delta }\frac{1}{\sigma }\phi (\frac{\mu }{\sigma })>0, \end{array}$$

which satisfies (14). The following proposition guanrantees the level of both the confidence interval and the hypothesis testing.

**Proposition 7** *If the marginal updated fiducial distribution of* *g*(*μ*, *σ*) *is an asymptotic confidence distribution. Then the level of the confidence interval is asymptotically* 1 − *α*. *The significance level of hypothesis testing is asymptotically α*.

The proof of Proposition 7 is given in S1 File.

From Propostion 7, if *g*(*μ*, *σ*) is taken as in Theorem 5 or Corollary 6, the confidence intervals in (8) and the p-values in (9) and (10) are asymptotically correct when *n* → ∞. When the sample size *n* is moderate, we give simulations in next section.

### 2.4 Sampling from the updated fiducial distribution

To give the confidence intervals of the parameters, we need to compute the *γ*-quantiles of the updated fiducial distributions. Similarly, to give the p-values of the hypothesis testing, we need to compute the cumumlative distribution functions of the marginal updated fiducial distirbutions at *g*_0_. However, it is difficult to give the closed forms of them. Fortunately, we can adopt a simple method to produce accurate sample from the updated fiducial distribution, which is known as the reject sampling method.

We can draw parameters from the “prior distribution” and accept the ones that generate the same number of zero as the observed data. This is similar to the reject-ABC method proposed first by [25, 26]. However, it shall be noticed that there is no approximation error in our sampling method for associated delta-lognormal distribution, since we don’t use summary statistics and accept only the parameters which generate the same number of zero. Thus the parameters we accepted are equavilent to sampling from the real posterior distribution (6).

Without loss of generality, assume first that the observation of sample size *n* is $x_{1},x_{2},\cdots ,x_{n_{1}},0,\cdots ,0$, where *x*_*i*_ > 0 for *i* = 1, ⋯, *n*_1_ and the rest *n*_0_ = *n* − *n*_1_ ones are zero. A log-transformation is then made to the nonzero observations $y_{1},y_{2},\cdots ,y_{n_{1}}$. Then the fiducial distributions of *μ* and *σ* is given by (3), which are

$$\begin{array}{c} \mu =\bar{y}-\frac{U}{V/\sqrt{n_{1}-1}}\frac{s}{\sqrt{n_{1}}},\;\sigma^{2}=\frac{\left(n_{1}-1\right)s^{2}}{V^{2}}, \end{array}$$ (15)

where *U* is the standard normal random variable while *V* is a *χ*^2^(*n*_1_ − 1) random variable.

1. Log-transformation is made on the nonzero observation, which is denote by $y_{1},y_{2},\cdots ,y_{n_{1}}$. The sample mean and sample variance are calculated and denoted by $\bar{y}$ and *s*^2^.
2. If *n*_1_ ≥ 2, sample *U* from the standard normal distribution and *V* from the $\chi_{n_{1}-1}^{2}$ distribution, respectively. To sample from the fiducial distribution of the parameters, we simply calculate *μ* and *σ*^2^ using (15). If *n*_1_ < 2, we draw samples from (4).
3. Calculate *δ* = *δ*(*μ*, *σ*) and draw samples from a binomial distribution B(*n*, *δ*(*μ*, *σ*)). We accept the parameters if the number of zero equals to *n*_0_.
4. The process is repeated until we accept a certain number of parameters.

With the sample from the updated fiducial distribution, we then consider the inference on the scalar function *g*(*μ*, *σ*). We first assume that a certain number, say *N*, parameters are accepted using reject sampling method. We denote these parameters by (*μ*_1_, *σ*_1_), (*μ*_2_, *σ*_2_), ⋯, (*μ*_*i*_, *σ*_*i*_), ⋯, (*μ*_*N*_, *σ*_*N*_). For the function *G* = *g*(*μ*, *σ*), let *g*_*i*_ = *g*(*μ*_*i*_, *σ*_*i*_), *i* = 1, 2, ⋯, *N*.

#### Confidence interval

The confidence interval (8) of *g*(*μ*, *σ*) can be computed as follow. We sort $g_{i}^{\prime }s$ in ascending order

$$\begin{array}{c} g_{\left(1\right)}\leq g_{\left(2\right)}\leq \cdots \leq g_{\left(N\right)}. \end{array}$$

Then we take

$$\begin{array}{c} {\hat{g}}_{\frac{\alpha }{2}}=g_{\left(\left[N\cdot \frac{\alpha }{2}\right]\right)},\;{\hat{g}}_{1-\frac{\alpha }{2}}=g_{\left(\left[N\left(1-\frac{\alpha }{2}\right)\right]\right)}, \end{array}$$ (16)

where [*a*] is the largest integer not larger than number *a*.

#### Hypothesis testing

The first hypothesis is testing whether (*μ*, *σ*) is in a nondegenerate region. This means that the null hypothesis is (*μ*, *σ*) ∈ Ω_0_ where Ω_0_ ⊂ ℜ × ℜ^+^. To test this hypothesis, we simply calculate the ratio of (*μ*_*i*_, *σ*_*i*_) contatining in Ω_0_ as follow and denote this value as the *p*-value

$$\begin{array}{c} \hat{p}{=}^{\#}\left\{\left(\mu_{i},\sigma_{i}\right)\in \Omega_{0}\right\}/N, \end{array}$$

where ^#^*A* means the number of set A.

We also consider testing the null hypothesis *H*_0_ : *θ* = *θ*_0_ versus *H*_1_ : *θ* ≠ *θ*_0_. The *p*-value under the null hypothesis is then

$$\begin{array}{c} \hat{p}=2\times \text{min}\;\left\{{\hat{p}}_{-},{\hat{p}}_{+}\right\}, \end{array}$$

where

$$\begin{array}{c} {\hat{p}}_{-}=F_{\theta_{0}}^{UF}\left(g_{0}\right)=\frac{1}{N}\#\left\{\theta_{i}\leq \theta_{0}\right\},\;{\hat{p}}_{+}=1-{\hat{p}}_{-}. \end{array}$$

Thus we reject the null hypothesis when the *p*-value is not larger than a given level *α*.

## 3 Simulation study

In this section we illustrate the performance of our confidence intervals and hypothesis testing when the sample size is moderate. We take *δ*(*μ*, *σ*) = Φ((*a* − *μ*)/*σ*). Without loss of generality, we take *a* = 0. Otherwise, for nonzero observation *X*_*i*_, let *Y*_*i*_ = *X*_*i*_*e*^−*a*^. Then log *Y*_*i*_ ∼ *N*(*μ*−*a*, *σ*^2^), which means that we take *μ* − *a* as the new location parameter. So we consider *δ*(*μ*, *σ*) = Φ(−*μ*/*σ*). We can sample from the updated fiducial distribution using the method we proposed. Three simulation studies are conducted in this section. The first simulation study shows the interval estimates of the parameters in associated delta-lognormal distribution, we compare this with that of the fiducial distributions to illustrate the improvements. In the second simulation study, we compare the estimates of population mean of the associated delta-lognormal and the traditional one when *δ* = 0.5. In the last simulation study, we focus on the estimation and hypothesis testing for *δ* in associated delta-lognormal distribution, we also compare the result with that of traditional one.

### 3.1 Simulation study I

**Fig 1:**
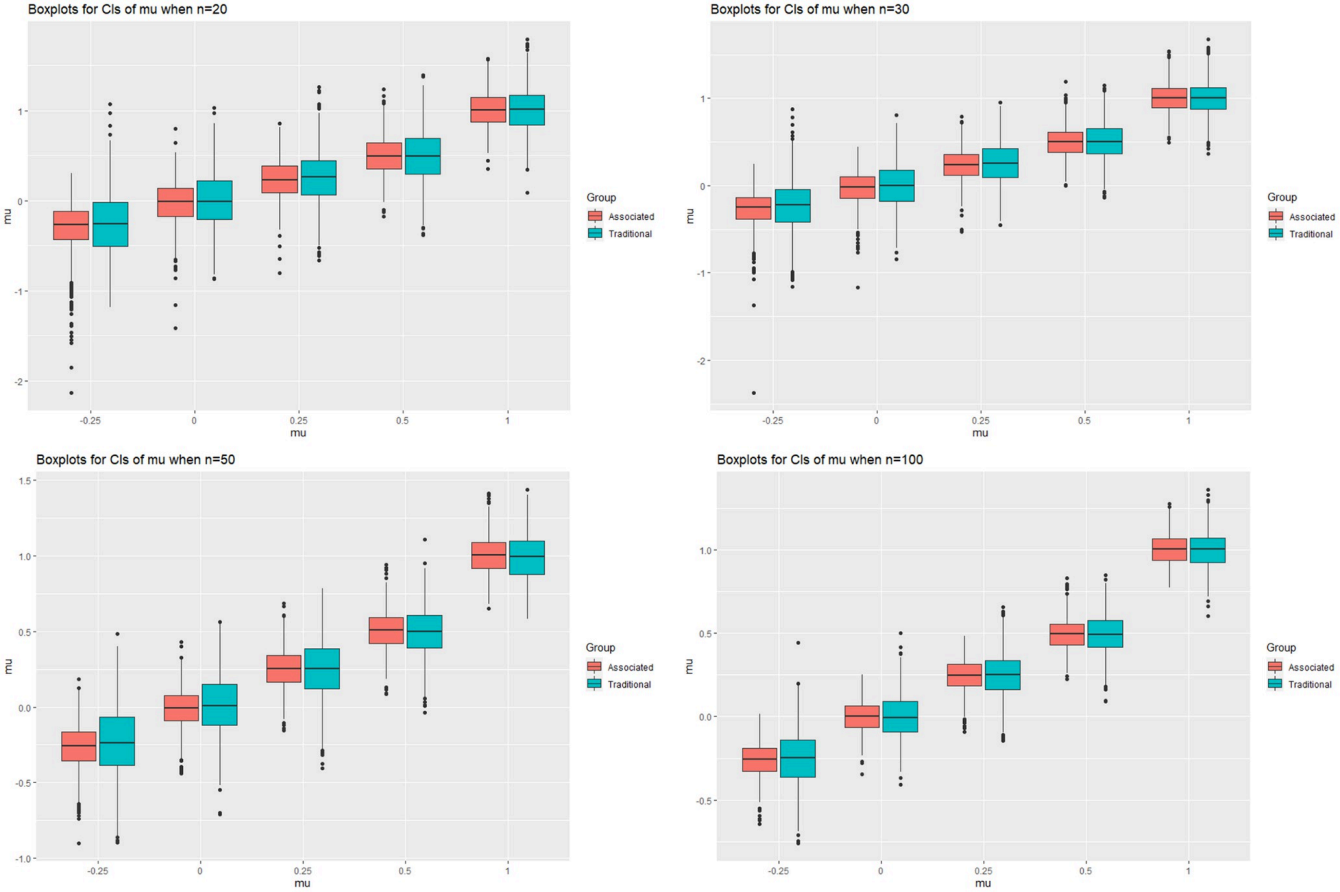
The confidence intervals of *μ* when *μ* = −0.25, 0, 0.25, 0.5, 1, *σ* = 0.5 and *n* = 20, 30, 50, 100.

**Fig 2:**
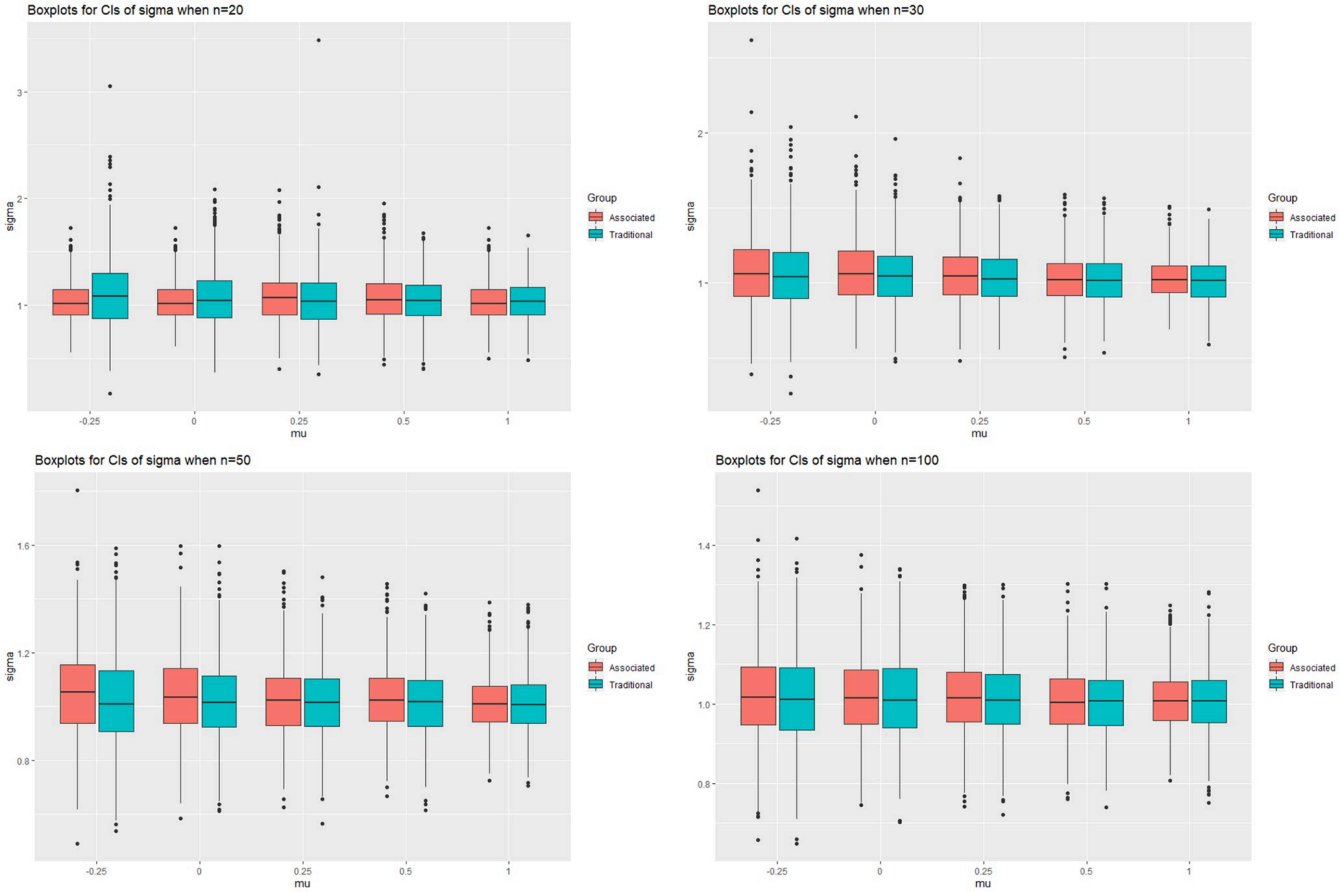
The confidence intervals of *σ* when *μ* = −0.25, 0, 0.25, 0.5, 1, *σ* = 0.5 and *n* = 20, 30, 50, 100.

In this simulation study we consider the estimate of *μ* and *σ*. The sample sizes considered are *n* = 20, 30, 50 and 100. We set the value of *σ* to 0.5, 1 and 2 while the value of *μ* is changed to make the corresponding *δ* = Φ(−*μ*/*σ*) approximately equal to 0.6, 0.5, 0.4, 0.3 and 0.15. Particularly, when *σ* = 1, the corresponding values of *μ* are −0.25, 0, 0.25, 0.5 and 1. For each parameter setting, we generate 1000 repetitions and for each one we sample *n* = 4000 pairs of (*μ*, *σ*) and calculate the 95 percent confidence intervals of *μ* and *σ* using the Eq (16). We compare the results with the estimates obtained from the fiducial distribution and the results are shown in the Figs 1 and 2, where the details are given in the Tables in S1 File. The figures are confidence intervals of *μ* and *σ* when *σ* = 1 and *n* = 20, 30, 50 and 100. The horizontal coordinates are the values of *μ* = −0.25, 0, 0.25, 0.5 and 1, while the vertical coordinates are the confidence intervals of *μ* or *σ* for different *μ*. The four plots from left to right and from top to bottom denote the cases when *n* = 20, 30, 50 and 100, respectively. The plot of *σ* = 1 and 2 are quite similar with that of 0.5, thus we don’t put the figures in our context.

For this specific *δ*, we can see that the estimate of *μ* is largely improved. The lower limits of *μ* become larger compare to the fiducial distribution while the upper limits are getting smaller. This leads to a significantly smaller confidence interval while retain the coverage probability. However, the impact on *σ* is not apparently as *μ*. The average length of the confidence intervals for *σ* generally get smaller than that of fiducial distribution, with the decreasing of *δ* and sample size *n*. The lower limits seems to be always bigger than that of the fiducial distribution while the upper limits gradually become smaller as *δ* and the sample size increase. We also notice that the distribution of *σ* is asymmetric, so we suggest to use the 2% and 97% quantile of the sampled *σ* to construct the 95% confidence interval of *σ*.

### 3.2 Simulation study II

In this simulation we consider the inference on the log population mean of the associated delta-lognormal distribution which has the form

$$\begin{array}{c} PM=\text{log}\;\left(1-\delta \left(\mu ,\sigma \right)\right)+\mu +\frac{1}{2}\sigma^{2}. \end{array}$$ (17)

**Fig 3:**
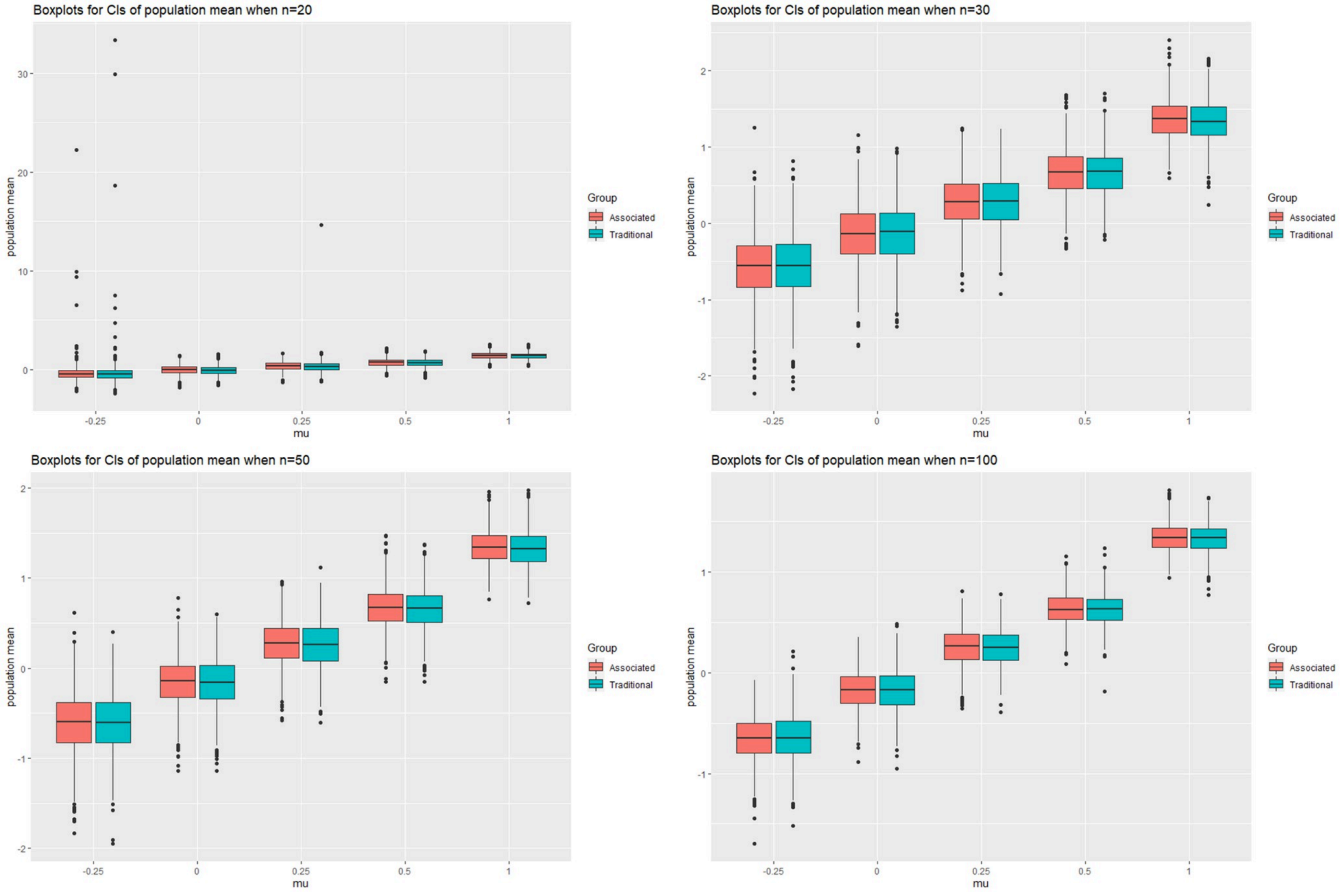
The confidence intervals of the population mean when *μ* = −0.25, 0, 0.25, 0.5, 1, *σ* = 1 and *n* = 20, 30, 50, 100.

The population mean of the delta lognormal distribution plays a crucial role in statistical analysis and inference. It is a measure of central tendency, providing a summary of the central location of the distribution. For example, in the real data of our paper, we estimate the diagnostic test charges of the patients. The true value of the parameters and the sample sizes are set as we did in the last simulation. We first consider the point estimate of the log population mean, the “posterior mean” and the “posterior median” are considered, the former is approximated by

$$\begin{array}{c} \hat{PM}\approx \frac{1}{N}\sum_{i=1}^{N}{PM}_{i}=\frac{1}{N}\sum_{i=1}^{N}g\left(\mu_{i},\sigma_{i}\right), \end{array}$$ (18)

while the latter is approximated by the 0.5 quantile of the *N* accepted values. We compute the mean bias and the mean squared error of these two estimates and compare with that of [14]. To obtain the estimate of Krishnamoorthy, we compute the mean of “Qtheta” in his paper. The result of the case when *σ* = 1 is shown in Table 1, the ones for *σ* = 0.5 and 2 can be found in the S1 File. “MB”, “MDB” and “GQB” stand for the mean bias of the posterior mean, posterior median and the estimate using the genralized quantity in [14]. “MSE” stands for the mean squared error and the subscripts indicate the three estimate. We also use Fig 3 for a better view of the two estimates. It should be noticed that some extreme cases may occur when *δ* is large and the sample size is small, as is shown in the first plot of Fig 3 where *n* = 20. In these extreme cases, there are only three or less nonzero observations, making the estimates far from the true value, thus the mean bias and mean squared error become meaningles. So we use the blanks to indicate such problem. However, we can see that the posterior median seems to be a better point estimate of the population mean. The mean bias and the mean squared error are generally smaller, especially when *σ* is large.

**Table 1:** Mean bias, mean squared error of the estimators of the population mean when *σ* = 1.

*μ*	*n*	MB	MSE_*M*_	MDB	MSE_*D*_	GQB	MSE_*G*_
−0.25	20	−−	−−	0.076	0.292	−−	−−
	30	0.093	0.192	0.049	0.175	0.079	0.206
	50	0.044	0.106	0.028	0.101	0.039	0.115
	100	0.001	0.050	−0.005	0.049	0.014	0.054
0	20	−−	−−	0.059	0.212	−−	−−
	30	0.077	0.150	0.041	0.139	0.060	0.152
	50	0.035	0.079	0.019	0.076	0.029	0.085
	100	0.019	0.041	0.013	0.040	0.008	0.042
0.25	20	0.123	0.208	0.058	0.181	0.078	0.195
	30	0.072	0.128	0.040	0.118	0.044	0.119
	50	0.038	0.066	0.023	0.063	0.021	0.067
	100	0.011	0.032	0.005	0.031	0.009	0.033
0.5	20	0.098	0.152	0.048	0.135	0.055	0.153
	30	0.066	0.097	0.038	0.090	0.034	0.097
	50	0.042	0.058	0.028	0.056	0.018	0.057
	100	0.016	0.026	0.010	0.025	0.009	0.026
1	20	0.066	0.117	0.028	0.107	0.042	0.111
	30	0.043	0.070	0.020	0.066	0.026	0.072
	50	0.021	0.040	0.011	0.032	0.014	0.041
	100	0.016	0.021	0.010	0.021	0.003	0.020

### 3.3 Simulation III

In this simulation we consider the case when *δ* = 0.5, which happens when *μ* = 0. We fix *μ* to 0 while *σ* = 0.5, 1 and 2. The sample sizes range from 20 to 100. We show in Table 2 the asymptotic 95% confidence intervals of *δ*. The estimate of *δ* is compared with that of the generalized fiducial distribution proposed by Hannig, which is a Beta distribution. It can be seen that the average length is smaller, which means that the estimate becomes more accurate. To illustrate this idea, we also test the hypothesis of *δ* = 0.1 to 0.9 for the case *δ* = Φ(−*μ*/*σ*) and calculate the *p*-value under the null hypothesis. In fact, we can consider any function of *μ* and *σ* after drawing pairs of parameters from the posterior distribution. The null hypothesis is set to *σ* = 0.1, 0.3, 0.5, 0.7 and 0.9. We calculate the *p*-value for both associated delta-lognormal and compare the result with the traditional one, which use the Beta distribution *Beta*(*n*_0_ + .5, *n*_1_ + .5) as the generalized fiducial distribution for *δ*. For each given set we generate 10000 samples and accept *N* = 10000 pairs of parameters. We calculate *p*_*i*_ = Φ(−*μ*_*i*_/*σ*_*i*_) for *i* = 1 to *N* and calculate the *p*-value for *δ* = *p*_0_, which is

$$\begin{array}{c} \hat{p}=2\times \text{min}\;\left[\#\left(p_{i}>p_{0}\right),\#\left(p_{i}<p_{0}\right)\right]. \end{array}$$

**Table 2:** Coverage probability, lower limit and upper limit for *δ* = 0.5.

*σ*	*n*	*ll*_*A*_	*ul*_*A*_	AL_*A*_	%	*ll*_*F*_	*ul*_*F*_	AL_*F*_
0.5	20	.347	.664	.317	95.24	.384	.789	.405
	30	.374	.637	.263	95.43	.298	.641	.343
	40	.389	.617	.228	94.91	.522	.804	.282
	50	.399	.604	.205	94.72	.403	.673	.270
	100	.428	.574	.146	95.69	.452	.645	.192
1	20	.346	.664	.318	95.21	.251	.662	.411
	30	.373	.635	.262	95.69	.241	.578	.337
	40	.389	.617	.228	95.07	.471	.762	.291
	50	.401	.605	.205	95.45	.346	.616	.270
	100	.428	.574	.146	95.28	.394	.587	.193
2	20	.346	.663	.317	95.12	.251	.662	.411
	30	.374	.637	.263	95.05	.455	.787	.332
	40	.390	.619	.229	95.88	.471	.762	.291
	50	.401	.605	.205	95.46	.346	.616	.270
	100	.430	.576	.146	94.87	.433	.626	.193

The result is shown in Table 3. A and D in the column named method represent the associated delta-lognormal distribution and the traditional one, respectively. We can see that the *p*-value of the same null hypothesis for associated delta-lognormal is more centralized than the traditional delta-lognormal distribution. This means that we are more likely to reject the null hypothesis of the associated delta-lognormal than the traditional ones when the null hypothesis is false.

**Table 3:** *p*-value of null hypothesis *δ* = *p*_0_.

*σ*	*n*	method	*p*_0_ = 0.1	*p*_0_ = 0.3	*p*_0_ = 0.5	*p*_0_ = 0.7	*p*_0_ = 0.9
0.5	20	A	.000	.057	.495	.101	.000
		D	.003	.173	.507	.169	.002
	30	A	.000	.027	.502	.042	.000
		D	.001	.094	.496	.110	.000
	40	A	.000	.010	.497	.200	.000
		D	.000	.064	.495	.065	.000
	50	A	.000	.003	.495	.010	.000
		D	.000	.039	.504	.031	.000
1	20	A	.000	.058	.500	.109	.001
		D	.003	.177	.506	.160	.002
	30	A	.000	.025	.495	.043	.000
		D	.000	.102	.511	.102	.000
	40	A	.000	.010	.490	.019	.000
		D	.000	.060	.498	.067	.000
	50	A	.000	.003	.510	.010	.000
		D	.000	.040	.508	.037	.000
2	20	A	.000	.067	.502	.097	.001
		D	.003	.171	.508	.170	.002
	30	A	.000	.025	.499	.042	.000
		D	.001	.105	.489	.098	.000
	40	A	.000	.010	.500	.018	.000
		D	.000	.060	.503	.062	.000
	50	A	.000	.003	.510	.009	.000
		D	.000	.038	.523	.033	.000

## 4 A real data example

In this section, we use the data set of diagnostic test charges in [27]’s study, see Table 4. This data set is analysed by [7], who showed that the postive part fit a lognormal distribution. The data set is further studied by [9, 14]. This data set contains 40 patients, but 10 of them had no diagnostic tests during the study period.

**Table 4:** Data set of the diagnostic test charges.

800	0	5733	0	0	9039	0	177
355	476	0	0	142	7790.9	0	967
1944	2010	964	255	0	4310	781	364
258	829	646	18081	0	681	177	3039
2569	139	1132	891	6432	140	425	0

We assume that the data set comes from an associated delta-lognormal population, where

$$\begin{array}{c} \delta \left(\mu ,\sigma \right)=\Phi (\frac{\text{log}\;x_{0}-\mu }{\sigma }). \end{array}$$

It can be calculated that $\hat{\mu }=6.854,\hat{\sigma }=1.367,x_{0}=376.657,\text{log}\;x_{0}=5.931$. We assume that the data are drawn from the associated delta-lognormal distribution below,

$$\begin{array}{c} G\left(x;\mu ,\sigma \right)=\{\begin{array}{cc} \delta (\mu ,\sigma ), & x=0,\\ \delta \left(\mu ,\sigma \right)+\left[1-\delta \left(\mu ,\sigma \right)\right]F_{LN}\left(x;\mu ,\sigma \right), & x>0, \end{array} \end{array}$$

where

$$\begin{array}{c} \delta \left(\mu ,\sigma \right)=\Phi (\frac{\text{log}\;x_{0}-\mu }{\sigma })=\Phi (\frac{5.931-\mu }{\sigma }). \end{array}$$

To test the goodness-of-fit, we choose *k* = 4 and create the partition, where *a*_1_, *a*_2_, *a*_3_, *a*_4_ are 250, 500, 900 and 3000, respectively. Given the level *α* = 0.05, the test statistic (7) is 3.916, which is smaller than $\chi_{0.95}^{2}\left(2\right)=5.991$. Thus the assumption of the model is accepted.

We give the confidence interval of the population mean using the method we proposed in this paper. We accept *N* = 10000 pairs of (*μ*, *σ*) and calculate the 2.5% and 97.5% quantiles. As we have mentioned in last section, the 2% and 97% quantiles are also considered since the distribution of *σ* is asymmetric. The result is compared with the Fiducial method proposed by [14] and the “MOVER” proposed by [16], see Table 5. It can be seen that the confidence interval is largely improved.

**Table 5:** The confidence interval of the population mean using different methods.

Method	CI	Length of CI
Fiducial	(987.8, 4654.2)	3666.4
MOVER	(981.1, 4573.3)	3592.2
CI (2.5%-97.5%)	(1002.2, 4578.4)	3576.2
CI (2%-97%)	(973.4, 4380.7)	3407.3

## 5 Results and discussion

In this paper, we consider the associated delta-lognormal distribution in which *δ* is associated to the location and scale parameters of the lognormal distribution. To combine the information in lognormal distribution with the discrete binomial distribution, we propose the updated fiducial distribution. We established the result that the confidence interval has asymtotically correct level while the significance level of the hypothesis testing is also asymtotically correct. To obtain the confidence intervals and the p-values, we suggest to use a rejection sampling motivated by approximate Bayesian computation to sample from the distributions. The “prior distribution” for *μ* and *σ* is chosen to be the fiducial distribution. The binomial likelihood function can be regarded as an updating to the fiducial distribution. We further infer on the functions of the parameters. We use a special case which is *δ* = Φ(−*μ*/*σ*) to illustrate our idea. We give the confidence interval of *μ* and *σ* for different sample sizes, and propose the method of testing the hypothesis for functions of *μ* and *σ*. For the cases when there are continuous and discrete data, we suggest first to obtain information from the continuous data. Such information are synthesized as a distribution, such as the fiducial distribution. The distribution is further updated by the discrete data through Bayes theorem. For further study, motivated by the research on delta-lognormal, see for example [14, 17, 28], difference or ratio between the parameters of two associated delta-lognormal distribution can be of interest as well as the quantile of the distribution.

## Supporting information

S1 File (ZIP)
