# Supplementary material for: Updated fiducial distribution of parameters in the associated delta-lognormal population
Source: PLoS One. 2024 Jun 5;19(6):e0298307. doi: 10.1371/journal.pone.0298307 (PMC11152293; doi:10.1371/journal.pone.0298307)
Supplement: S1 File — (ZIP) [file pone.0298307.s001.zip › Supplementary/Supplementary material.pdf]

SUPPLEMENTARY MATERIAL

YUFAN WANG AND XINGZHONG XU

Appendix

Proof of Proposition 1. The density function is

$$f(z,x;\mu,\sigma)=\delta^z(1-\delta)^{1-z}\Big[\frac{1}{\sqrt{2\pi}\sigma}\exp\big(-\frac{(\log x-\mu)^2}{2\sigma^2}\big)/\phi(\log x)\Big]^{1-z}.$$

Here for convience we abbreviate $\delta(\mu,\sigma)$ to δ, but we keep in mind that it is a function of (μ,σ). When $z=1$, the density becomes $f(1,x;\mu,\sigma)=\delta$, the first order derivative of $\sqrt{f(1,x;\mu,\sigma)}$ is continuous by the assumptions on δ. When $z=0$, the density becomes

$$f(0,x;\mu,\sigma)=\frac{1-\delta}{\sigma}\frac{\phi(\frac{\log x-\mu}{\sigma})}{\phi(\log x)}.$$

Similarly the first order derivative of $\sqrt{f(0,x;\mu,\sigma)}$ is surely continuous in $\theta=(\mu,\sigma)$ for every x.

We then calculate the Fisher information matrix.

$$\log f(\mu,\sigma;z,x)=z\log\delta+(1-z)[\log(1-\delta)-\log(\sigma)-\frac{1}{2\sigma^2}(\log x-\mu)^2+\frac{1}{2}(\log x)^2].$$

Thus the derivatives can be given as

$$\begin{aligned}\frac{\partial l}{\partial\mu}&=\frac{z}{\delta}\frac{\partial\delta}{\partial\mu}+(1-z)\Big(-\frac{1}{1-\delta}\frac{\partial\delta}{\partial\mu}+\frac{\log x-\mu}{\sigma^2}\Big)\\ \frac{\partial l}{\partial\sigma}&=\frac{z}{\delta}\frac{\partial\delta}{\partial\sigma}+(1-z)\Big[-\frac{1}{1-\delta}\frac{\partial\delta}{\partial\sigma}-\frac{1}{\sigma}+\frac{(\log x-\mu)^2}{\sigma^3}\Big].\end{aligned}$$

Since $\log X\sim N(\mu,\sigma^2)$, we have

$$E_\theta\Big(\frac{\log X-\mu}{\sigma}\Big)^2=1,\quad E_\theta\Big[\frac{(\log X-\mu)^2}{\sigma^2}-1\Big]^2=2.$$

Furthermore, for the variable Z, we have

$$E_\theta(Z)=E_\theta(Z^2)=\delta,\quad E_\theta(1-Z)=E_\theta(1-Z)^2=1-\delta,\quad E_\theta[Z(1-Z)]=0.$$

Then we obtain

$$\begin{aligned}I_{11}(\theta)=E_\theta\Big(\frac{\partial f}{\partial\mu}\Big)^2&=E_\theta\Big[\frac{Z^2}{\delta^2}\Big(\frac{\partial\delta}{\partial\mu}\Big)^2+\frac{2Z(1-Z)}{\delta}\frac{\partial\delta}{\partial\mu}\Big(-\frac{1}{1-\delta}\frac{\partial\delta}{\partial\mu}+\frac{\log X-\mu}{\sigma^2}\Big)\\&\quad+(1-Z)^2\Big(-\frac{1}{1-\delta}\frac{\partial\delta}{\partial\mu}+\frac{\log X-\mu}{\sigma^2}\Big)^2\Big]\\&=\frac{1}{\delta}\Big(\frac{\partial\delta}{\partial\mu}\Big)^2+(1-\delta)E_\theta\Big(-\frac{1}{1-\delta}\frac{\partial\delta}{\partial\mu}+\frac{\log X-\mu}{\sigma^2}\Big)^2\\&=\frac{1}{\delta}\Big(\frac{\partial\delta}{\partial\mu}\Big)^2+\frac{1}{1-\delta}\Big(\frac{\partial\delta}{\partial\mu}\Big)^2+\frac{1-\delta}{\sigma^2}\\&=\frac{1}{\delta(1-\delta)}\Big(\frac{\partial\delta}{\partial\mu}\Big)^2+\frac{1-\delta}{\sigma^2}.\end{aligned}$$

Similarly we obtain

$$I_{22}(\theta) = \frac{1}{\delta(1-\delta)}\Big(\frac{\partial\delta}{\partial\sigma}\Big)^2 + \frac{1-\delta}{\sigma^2}\times 2,$$
$$I_{12}(\theta) = \frac{1}{\delta(1-\delta)}\frac{\partial\delta}{\partial\mu}\frac{\partial\delta}{\partial\sigma}.$$

Hence the Fisher information matrix is

$$\begin{aligned} I(\theta) &= \begin{pmatrix} \frac{1}{\delta(1-\delta)}\Big(\frac{\partial\delta}{\partial\mu}\Big)^2 + \frac{1-\delta}{\sigma^2} & \frac{1}{\delta(1-\delta)}\frac{\partial\delta}{\partial\mu}\frac{\partial\delta}{\partial\sigma} \\ \frac{1}{\delta(1-\delta)}\frac{\partial\delta}{\partial\mu}\frac{\partial\delta}{\partial\sigma} & \frac{1}{\delta(1-\delta)}\Big(\frac{\partial\delta}{\partial\sigma}\Big)^2 + \frac{2-2\delta}{\sigma^2} \end{pmatrix} \\ &= \begin{pmatrix} \frac{1-\delta}{\sigma^2} & 0 \\ 0 & \frac{2-2\delta}{\sigma^2} \end{pmatrix} + \frac{1}{\delta(1-\delta)}\begin{pmatrix} \Big(\frac{\partial\delta}{\partial\mu}\Big)^2 & \frac{\partial\delta}{\partial\mu}\frac{\partial\delta}{\partial\sigma} \\ \frac{\partial\delta}{\partial\mu}\frac{\partial\delta}{\partial\sigma} & \Big(\frac{\partial\delta}{\partial\sigma}\Big)^2 \end{pmatrix}. \end{aligned} \tag{1}$$

Therefore under the assumption of the proposition, the Fisher matrix is nondegenerate. Then by Definition 1 and Lemma 1, the conclusion holds.

□

Proof of Theorem 1. The density functions $f(z, x; \mu, \sigma)$ are quadratic mean differentiable by Proposition 1. Further, we show that the Fisher information matrix is nondegenerate in the proof of Proposition 1. Then we only need to prove that for every $\varepsilon > 0$ there exists a sequence of test ψ_n such that

$$E^n_{\theta_0}\psi_n(X) \to 0, \qquad \sup_{\|\theta-\theta_0\|\geq\varepsilon} E^n_\theta[1-\psi_n(X)] \to 0.$$

Let $\hat{\mu} = \bar{Y}$ and $\hat{\sigma^2} = S^2$, where $\bar{Y} = 0$ if $n_1 = 0$, and $S^2 = 1$ if $n_1 = 0, 1$. Let $\theta_0 = (\mu_0, \sigma_0)^T$ be the true value of the parameters.

We then take a sequence of test ψ_n as

$$\psi_n(x) = \begin{cases} 1, & n_1 = 0, 1 \quad or \quad \Big\{\sqrt{(\hat{\mu}-\mu_0)^2 + (\hat{\sigma^2}-\sigma_0^2)^2} \geq \varepsilon n_1^{-1/3}, n_1 > 1\Big\}, \\ 0, & else. \end{cases}$$

Then the expectation of the test can be calculated as

$$\begin{aligned} E^n_{\theta_0}(\psi_n(X)) &= P_{\theta_0}\big(N_1 = 0\big) + P_{\theta_0}\big(N_1 = 1\big) \\ &\quad + P_{\theta_0}\Big(\sqrt{(\hat{\mu}-\mu_0)^2 + (\hat{\sigma^2}-\sigma_0^2)^2} \geq \varepsilon N_1^{-1/3}, N_1 > 1\Big) \\ &\leq \delta^n + n\delta^{n-1}(1-\delta) + P\Big(|\hat{\mu}-\mu_0| \geq \frac{\varepsilon}{\sqrt{2}}N_1^{-1/3}, N_1 > 1\Big) \\ &\quad + P\Big(|\hat{\sigma^2}-\sigma_0^2| \geq \frac{\varepsilon}{\sqrt{2}}N_1^{-1/3}, N_1 > 1\Big). \end{aligned}$$

By the Chebyshev's inequality, we have

$$P_{\theta_0}\Big(|\hat{\mu}-\mu_0| \geq \frac{\varepsilon}{\sqrt{2}}N_1^{-1/3}, N_1 > 1\Big) \leq E^n_{\theta_0}\Big[\frac{2\sigma^2}{\varepsilon^2 N_1^{1/3}} I_{\{N_1>1\}}\Big]$$
$$P_{\theta_0}\Big(|\hat{\sigma^2}-\sigma_0^2| \geq \frac{\varepsilon}{\sqrt{2}}N_1^{-1/3}, N_1 > 1\Big) \leq E^n_{\theta_0}\Big[\frac{4\sigma^2}{\varepsilon^2 (N_1^{1/3} - N_1^{-2/3})} I_{\{N_1>1\}}\Big].$$

Thus

$$P_{\theta_0}\Big(|\hat{\mu}-\mu_0| \geq \frac{\varepsilon}{2}N_1^{-1/3}, N_1 > 1\Big) + P_{\theta_0}\Big(|\hat{\sigma^2}-\sigma_0^2| \geq \frac{\varepsilon}{2}N_1^{-1/3}, N_1 > 1\Big) \leq E^n_{\theta_0}\Big(\frac{10\sigma^2}{\varepsilon^2 N_1^{1/3}}, N_1 > 1\Big).$$

When $n \to +\infty$, $N_1/n \xrightarrow{P_{\theta_0}} 1-\delta$. Thus by Lebesgue dominating convergence theorem,

$$E^n_{\theta_0}(N_1^{-\frac{1}{3}}) = E^n_{\theta_0}\Big(n^{-\frac{1}{3}}(\frac{N_1}{n})^{-\frac{1}{3}}\Big) \to 0.$$

We have proven that $E^n_{\theta_0}(\psi_n) \to 0$.

For $E^n_\theta[1-\psi_n(X)]$ we have

$$\begin{aligned}
E^n_\theta[1-\psi_n(X)] &= P_\theta\Big(\|\hat\theta-\theta_0\| < \varepsilon N_1^{-1/3}, N_1 > 1\Big) \\
&\le P_\theta\Big(\|\theta-\theta_0\| - \|\hat\theta-\theta\| < \varepsilon N_1^{-1/3}, N_1 > 1\Big) \\
&\le P_\theta\Big(\|\hat\theta-\theta\| > \|\theta-\theta_0\| - \frac{\varepsilon}{2}, N_1 > 1\Big) \\
&\le \frac{E^n_\theta\big(\|\hat\theta-\theta\|^2 I_{\{N_1>1\}}\big)}{\big(\|\theta-\theta_0\|-\frac{\varepsilon}{2}\big)^2} \\
&= \frac{1}{\big(\|\theta-\theta_0\|-\frac{\varepsilon}{2}\big)^2}\Big[E^n_\theta\big(\frac{\sigma^2}{N_1} + \frac{2\sigma^4}{N_1-1}\big)I_{\{N_1>1\}}\Big] \\
&\le \frac{\sigma^2+4\sigma^4}{\big(\|\theta-\theta_0\|-\frac{\varepsilon}{2}\big)^2} E^n_\theta\Big(\frac{1}{N_1}I_{\{N_1>1\}}\Big).
\end{aligned}$$

It can be seen that the fraction is bounded. For the expectation we have

$$\begin{aligned}
E_\theta\Big(\frac{1}{N_1}I_{\{N_1>1\}}\Big) &< E_\theta\Big(\frac{2}{N_1+1}I_{\{N_1>1\}}\Big) \\
&= 2\sum_{n_1=2}^{n}\frac{1}{n_1+1}\binom{n}{n_1}(1-\delta)^{n_1}\delta^{n-n_1} \\
&= 2\sum_{n_1=2}^{n}\frac{n!}{(n_1+1)!(n-n_1)!}(1-\delta)^{n_1}\delta^{n-n_1} \\
&= \frac{2}{n+1}\sum_{i=3}^{n+1}\frac{(n+1)!}{i!(n+1-i)!}(1-\delta)^{i-1}\delta^{n+1-i} \\
&= \frac{2}{n+1}\frac{1}{1-\delta}\sum_{i=3}^{n+1}\binom{n+1}{i}(1-\delta)^{i}\delta^{n+1-i} \\
&= \frac{2}{n+1}\Big\{\frac{1}{1-\delta}\Big[1-\delta^{n+1}-(n+1)(1-\delta)\delta^n - \frac{n(n+1)}{2}(1-\delta)^2\delta^{n-1}\Big]\Big\} \\
&\triangleq \frac{2}{n+1}h(\delta).
\end{aligned}$$

With simple calculation we can get

$$\lim_{\delta\to0}h(\delta)\to1, \quad \lim_{\delta\to1}h(\delta)\to0.$$

This means that $h(\delta)$ is also bounded. Thus

$$\sup_{\|\theta-\theta_0\|>\varepsilon} E^n_\theta[1-\psi_n(X)] \le B\frac{1}{n+1},$$

where B is a bounded value which is not related to μ and σ. This indicates that

$$\sup_{\|\theta-\theta_0\|\ge\varepsilon} E^n_\theta[1-\psi_n(Y)] \to 0.$$

Since $1/\sigma$ is absolutely continuous in a neighborhood of $\theta_0 = (\mu_0, \sigma_0)$ with a continuous positive density at θ_0. The proof is accomplished. □

Proof of Theorem 2. Let $g_0 = g(\mu_0, \sigma_0)$. Then

$$\begin{aligned} F_G^{UF}(g_0|X_{obs}) &= P^{UF}(g(\mu,\sigma) \le g_0) \\ &= P^{UF}\Big(K(a\mu + b\sigma) \le K(a\mu_0 + b\sigma_0)\Big) \\ &= P^{UF}\Big(a\mu + b\sigma \le a\mu_0 + b\sigma_0\Big) \\ &= P^{UF}\Big(a(\mu - \mu_0) + b(\sigma - \sigma_0) \le 0\Big) \\ &= P^{UF}\Big((a,b)(\theta - \theta_0) \le 0\Big), \end{aligned}$$

where P^{UF} is the probability measure corresponding to the updated fiducial distribution. Let $\sqrt{n}(\theta - \theta_0) = \tau$. Then by Theorem 1, we have

$$\Big|P^{UF}\Big((a,b)(\theta-\theta_0) \le 0\Big) - P^N\Big((a,b)\tau \le 0\Big)\Big| \overset{P^n_{\theta_0}}{\to} 0, \tag{2}$$

where P^N is the probability measure of the normal distribution $N(\Delta_{n,\theta_0}, I^{-1}(\theta_0))$, which the two-dimensional random vector τ obeys. Hence

$$(a,b)\tau \sim N\big((a,b)\Delta_{n,\theta_0}, (a,b)I^{-1}_{\theta_0}(a,b)^T\big)$$

under P^N. Thus

$$P^N\big((a,b)\tau \le 0\big) = \Phi\Big(\frac{-(a,b)\Delta_{n,\theta_0}}{\sqrt{(a,b)I^{-1}_{\theta_0}(a,b)^T}}\Big). \tag{3}$$

Because

$$\Delta_{n,\theta_0} \overset{P_{\theta_0}}{\rightsquigarrow} N(0, I^{-1}(\theta_0)),$$

therefore

$$\Phi\Big(\frac{-(a,b)\Delta_{n,\theta_0}}{\sqrt{(a,b)I^{-1}_{\theta_0}(a,b)^T}}\Big) \overset{P_{\theta_0}}{\rightsquigarrow} U(0,1). \tag{4}$$

Combine with (2),(3) and (4) we can get

$$F^{UF}(g_0) \overset{P_{\theta_0}}{\rightsquigarrow} U(0,1).$$

□

Proof of Corollary1. It is the direct consequence of Theorem 2 for $g_i(\theta), i = 1,2,3$.

For the random variable $G = \Phi[(\log x_0 - \mu)/\sigma]$, we have

$$\begin{aligned} P^{UF}(G \le g_0) &= P^{UF}\Big(\Phi\big(\frac{\log x_0 - \mu}{\sigma}\big) \le \Phi\big(\frac{\log x_0 - \mu_0}{\sigma_0}\big)\Big) \\ &= P^{UF}\Big(\frac{\log x_0 - \mu}{\sigma} \le \frac{\log x_0 - \mu_0}{\sigma_0}\Big) \\ &= P^{UF}\big(\sigma_0 \log x_0 - \mu\sigma_0 \le \sigma \log x_0 - \sigma\mu_0\big) \\ &= P^{UF}\big(\sigma_0\mu + (\log x_0 - \mu_0)\sigma \ge \sigma_0 \log x_0\big). \end{aligned}$$

Let $g^*(\mu,\sigma) = \sigma_0\mu + (\log x_0 - \mu_0)\sigma$. We can see that $g^*(\mu,\sigma)$ satisfies the conditions of Theorem 2 and

$$g^*(\mu_0,\sigma_0) = \sigma_0\mu_0 + (\log x_0 - \mu_0)\sigma_0 = \log x_0\sigma_0.$$

This means that

$$P^{UF}\big(g^*(\mu,\sigma) \le g^*(\mu_0,\sigma_0)\big) \rightsquigarrow U(0,1).$$

So we can get

$$P^{UF}(G \le g_0) = P^{UF}\big(g^*(\mu,\sigma) \ge g^*(\mu_0,\sigma_0)\big) \rightsquigarrow U(0,1).$$

Finally we consider the log population mean. Let

$$\mu^* = \log M = \log(1 - \delta(\mu, \sigma)) + \mu + \frac{1}{2}\sigma^2.$$

A reparameterization is made on (μ, σ) to (μ^*, σ), which is well defined when

$$\frac{\partial \mu^*}{\partial \mu} = \frac{1}{1-\delta}\Big(-\frac{\partial \delta}{\partial \mu}\Big) + 1 > 0.$$

Then the density function $f(z, x; \mu, \sigma)$ can be rewritten as $f^*(z, x; \mu^*, \sigma)$, under which Theorem 2 also holds. Then by (i) in Corollary 1, the marginal updated fiducial distribution of μ^* is an asymptotic confidence distribution. □

Proof of Proposition 2. First we can show that

$$\begin{aligned} P_{\theta_0}(g_0 \le \hat{g}_\gamma) &= P_{\theta_0}\big(F_G^{UF}(g_0) \le F_G^{UF}(\hat{g}_\gamma)\big) \\ &= P_{\theta_0}\big((F_G^{UF}(g_0) \le \gamma\big) \\ &\to \gamma. \end{aligned} \tag{5}$$

This means $P_{\theta_0}(g \ge \hat{g}_\gamma) \to 1 - \gamma$. Then the confidence interval with level $1 - \alpha$ satisfies

$$\begin{aligned} P_{\theta_0}(\hat{g}_{\frac{\alpha}{2}} \le g \le \hat{g}_{1-\frac{\alpha}{2}}) &= 1 - P_{\theta_0}(g \le \hat{g}_{\frac{\alpha}{2}}) - P_{\theta_0}(g \ge \hat{g}_{1-\frac{\alpha}{2}}) \\ &= 1 - \frac{\alpha}{2} - \Big(1 - \big(1 - \frac{\alpha}{2}\big)\Big) \\ &= 1 - \alpha. \end{aligned}$$

For the one-sided hypothesis, the conclusion surely holds by using (5). For the two-sided hypothesis, we have

$$\begin{aligned} P_{\theta_0}(\hat{p} \le \alpha) &= P_{\theta_0}(2\min\{\hat{p}, 1-\hat{p}\} \le \alpha) \\ &= P_{\theta_0}\Big(\min\{\hat{p}, 1-\hat{p}\} \le \frac{\alpha}{2}\Big) \\ &= P_{\theta_0}\Big(\hat{p} \le \frac{\alpha}{2} \quad or \quad \hat{p} \ge 1 - \frac{\alpha}{2}\Big) \\ &= P_{\theta_0}\Big(\hat{p} \le \frac{\alpha}{2}\Big) + P_{\theta_0}\Big(\hat{p} \ge 1 - \frac{\alpha}{2}\Big) \\ &\to \frac{\alpha}{2} + \frac{\alpha}{2} = \alpha. \end{aligned}$$

□
